# Synergistic efficacy and safety of PD-1/PD-L1 inhibitors combined with nab-paclitaxel and platinum chemotherapy in NSCLC: A systematic review and meta-analysis of randomized controlled trials

**DOI:** 10.3389/fonc.2025.1649777

**Published:** 2025-12-03

**Authors:** Yijing Shen

**Affiliations:** School of Pharmacy, Yanbian University, Yanji, China

**Keywords:** non-small cell lung cancer, PD-1/PD-L1 inhibitors, nab-paclitaxel, platinum-based chemotherapy, meta-analysis, immune-related adverse events

## Abstract

**Background:**

Non-small cell lung cancer (NSCLC), the leading cause of cancer mortality, often requires platinum-based chemotherapy. Nanoparticle albumin-bound paclitaxel (nab-paclitaxel) improves drug delivery, while PD-1/PD-L1 inhibitors enhance antitumor immunity. Preclinical studies suggest synergy, but clinical evidence for combining these agents remains limited.

**Methods:**

Following PRISMA guidelines, this meta-analysis pooled data from four randomized controlled trials (1998 patients). Databases (PubMed, Embase, Cochrane Central, web of science) were searched until January 2025. Trials comparing PD-1/PD-L1 inhibitors + nab-paclitaxel-platinum (experimental) versus chemotherapy alone (control) were included. Outcomes included including objective response rate (ORR), progression-free survival (PFS), overall survival (OS), pathologic complete response (pCR), major pathologic response (MPR), and grade ≥3 adverse events (AEs). Statistical analyses used fixed/random-effects models.

**Results:**

The combination therapy significantly improved ORR (OR = 1.81, 95% CI: 1.49–2.20, *p* < 0.001), PFS (HR = 0.65, 95% CI: 0.58–0.73), and OS (HR = 0.81, 95% CI: 0.72–0.91). In the resectable setting, neoadjuvant treatment resulted in higher pathologic complete response (pCR: 32.6% vs. 8.9%) and major pathologic response (MPR: 65.1% vs. 15.6%). Subgroup analyses showed enhanced benefit in PD-L1-high patients. The experimental group had increased risk of grade ≥3 thrombocytopenia (RR = 1.83) and immune-related adverse events (RR = 2.49).

**Conclusion:**

PD-1/PD-L1 inhibitors combined with nab-paclitaxel-platinum enhance survival outcomes in NSCLC, particularly for PD-L1-high patients. Despite increased immune-related toxicity risks, this regimen represents a promising first-line option, warranting biomarker-driven selection and vigilant AE management. Future studies should address heterogeneity in platinum agents and optimize patient stratification.

## 1 Introduction

Lung cancer persists as the foremost contributor to cancer-related mortality worldwide, with non-small cell lung cancer (NSCLC) constituting approximately 85% of all diagnosed cases (1). Globally, an estimated 2.2 million new lung cancer diagnoses and 1.8 million deaths were reported in 2020 alone, highlighting the critical need for innovative and effective therapeutic interventions (2). Despite advancements in early detection methods and molecularly targeted therapies, a significant proportion of NSCLC patients present with advanced or metastatic disease at initial diagnosis, necessitating reliance on systemic chemotherapy as a cornerstone of treatment (3).

Historically, platinum-based doublets, such as cisplatin or carboplatin combined with taxanes like paclitaxel or pemetrexed, have served as the backbone of first-line therapy for NSCLC (4). Among taxanes, paclitaxel has been extensively utilized; however, its solvent-based formulation, which relies on polyethoxylated castor oil as a delivery vehicle, is frequently associated with hypersensitivity reactions and suboptimal tumor penetration due to poor biodistribution (5). The advent of nanotechnology in oncology drug delivery catalyzed the development of nanoparticle albumin-bound paclitaxel (nab-paclitaxel), a formulation designed to circumvent these limitations by leveraging endogenous albumin pathways for enhanced tumor-specific accumulation (6). In a landmark phase III clinical trial, nab-paclitaxel combined with carboplatin demonstrated superior objective response rates and a reduced incidence of neuropathy compared to its solvent-based counterpart, ultimately securing FDA approval as a first-line regimen for NSCLC (7).

The therapeutic paradigm for NSCLC has undergone further transformation with the integration of immune checkpoint inhibitors (ICIs), particularly those targeting the PD-1/PD-L1 axis. These agents disrupt critical immune evasion mechanisms employed by tumors, thereby restoring antitumor immunity and eliciting durable clinical responses in a subset of patients (8). Pivotal clinical trials, including KEYNOTE-189 and IMpower130, established the efficacy of combining ICIs such as pembrolizumab and atezolizumab with platinum-pemetrexed chemotherapy, demonstrating statistically significant improvements in overall survival (OS) and progression-free survival (PFS) compared to chemotherapy alone (9, 10). Despite these advances, the potential synergy between ICIs and nab-paclitaxel-platinum combinations remains inadequately explored. Preclinical studies suggest that nab-paclitaxel may exert immunomodulatory effects, such as enhancing dendritic cell activation and promoting tumor antigen presentation, which could synergize with PD-1/PD-L1 blockade to amplify antitumor responses (11). Early-phase clinical trials, exemplified by IMpower132, have reported promising outcomes with atezolizumab in combination with nab-paclitaxel and carboplatin, achieving a median PFS of 7.0 months compared to 5.7 months with chemotherapy alone (12).

Despite these advances, no comprehensive synthesis has evaluated the efficacy and safety of PD-1/PD-L1 inhibitors combined with nab-paclitaxel and platinum across multiple trials. Existing meta-analyses predominantly focus on solvent-based taxanes or pemetrexed-based regimens (13, 14). Our study addresses this gap by conducting a systematic review and meta-analysis of four randomized controlled trials (RCTs), aiming to quantify the clinical benefits and risks of this triplet therapy. By synthesizing data from 1998 patients, our findings offer valuable evidence to help guide clinicians in optimizing first-line treatment for NSCLC.

## 2 Methods

This report was designed and executed in accordance with the PRISMA (Preferred Reporting Items for Systematic Reviews and Meta-Analyses) (15, 16). As the study exclusively utilized aggregated data from publicly available published literature, ethical committee approval and individual patient consent were deemed unnecessary under current research ethics guidelines.

### 2.1 Literature search

Aiming to find as many trials as possible that might fit our study topic, mainstay medical databases were searched, including PubMed, Embase, Web of Science, the Cochrane Central Register of Controlled Trials, from inception to January 24, 2025. Further, a two-person independent screening was throughout the search and literature screening session to control for research bias, and a third person resolved the disagreement between the two. The indexed terms albumin bound paclitaxel, Carcinoma, Non-Small-Cell Lung, Lung Neoplasms, Immune Checkpoint Inhibitors and the corresponding free-text terms with them were searched. Upon review of the literature, available Immune Checkpoint Inhibitors and related trade names and product development codes were included. The specific search strategy was described in **Supplementary Material 1**. Although a detailed internal protocol was developed *a priori*, it was not prospectively registered in a public registry such as PROSPERO.

### 2.2 Selection criteria

Eligible studies fulfilled the following requirements were included: (1) Participants were diagnosed with NSCLC through histological or pathological confirmation; (2) Comparative evaluation of therapeutic regimens, where the experimental group received albumin-bound paclitaxel combined with PD-1/PD-L1 inhibitors and platinum-based agents, while the control group received platinum-based therapy combined with albumin-bound paclitaxel; We included studies across all disease stages (metastatic and resectable) to comprehensively assess the regimen’s utility; (3) Reported outcomes included progression-free survival (PFS), overall survival (OS), Event-Free Survival (EFS), Disease-Free Survival(DFS), objective response rate (ORR), pathologic complete response (pCR), major pathologic response (MPR) and grade ≥3 adverse events (AEs); (4) The study category was clinical trial or prospective study, and the study was randomized and controlled.

Exclusion criteria were as follows (1) Studies that met the inclusion criteria but lacked randomization or were non-controlled; (2) duplicate publications or overlapping datasets; (3) full text unavailable or lacking accessible outcome data necessary for meta-analysis; (4) articles published as reviews, meta-analyses, letters, commentaries, case reports, or conference abstracts without subsequent full-length publication.

### 2.3 Data extraction

Selected publications were screened and information extraction was performed by both authors, with two categories of information extracted (1) basic study information, including the name and year of publication of the first author; median number of patients, gender, and age; squamous cell carcinoma (SCC)/non-SCC; disease stage; and study style (**Table 1**). (2) Study treatment and outcome information, including treatment regimen per group; study sample size; treatment duration; primary outcome points (including PFS, OS, ORR, EFS, DFS, pCR and MPR); and secondary outcome points (including number of AE ≥ grade 3) (**Table 2**). In case of disagreement, consensus was reached by discussion with the third author.

**Table 1:** The characteristics of the included comparative studies.

References	Years	Age median (range)		Gender (male/female)		PD-1/PD-L1	Design style	SCC/non-SCC	
		EG	CG	EG	CG			EG	CG
Lei et al. (17)	2023	61 (54-65)	61 (54-65)	34/9	40/5	Camrelizumab (PD-1)	RCT	27/15	32/11
Jotte et al.	2018	65 (23-83)	65 (38-86)	280/63	277/63	Atezolizumab (PD-L1)	RCT	343/0	340/0
West et al. (10)	2019	64 (18-86)		266/185	134/92	Atezolizumab (PD-L1)	RCT	0/451	0/226
Zhou et al. (18)	2024	63 (41-81)	63.0 (35-86)	321/37	167/12	Serplulimab (PD-1)	RCT	358/0	179/0

EG, control; CG, experimental; SCC, Squamous cell carcinoma.

**Table 2:** Interventions and outcome indicators of the included studies.

References	Phase	Stage	Number		Interventions		Duration	Indicators
			EG	CG	EG	CG		
Lei et al. (17)	Phase II	IIIA/IIIB	43	45	Camrelizumab 200 mg (day 1) + nab-paclitaxel 130 mg/m² (day 2 to day 8) + platinum (cisplatin 75 mg/m², carboplatin AUC 5, or nedaplatin 100 mg/m²)	Nab-paclitaxel 130 mg/m² (day 2 to day 8) + platinum (cisplatin 75 mg/m², carboplatin AUC 5, or nedaplatin 100 mg/m²)	q3w, 3 cycles	EFS, DFS, pCR, MPR, ORR
Jotte et al.	Phase III	IV	343	340	Atezolizumab 1200 mg (q3w) + carboplatin AUC 6 (q3w) + nab-paclitaxel 100 mg/m²	carboplatin AUC 6 (q3w) + nab-paclitaxel 100 mg/m²	q3w, (4–6 cycles)	PFS, OS, ORR
West et al. (10)	Phase IIIB	IV	451	228	Atezolizumab 1200 mg (q3w) + carboplatin AUC 6 (q3w) + nab-paclitaxel 100 mg/m²	carboplatin AUC 6 (q3w) + nab-paclitaxel 100 mg/m²	q3w, (4–6 cycles)	PFS, OS, ORR
Zhou et al. (18)	Phase III	IIIB/IIIC/IV	358	179	Serplulimab 4.5 mg/kg (q3w) + nab-paclitaxel 100 mg/m² (days 1/8/15) + carboplatin AUC 5–6 (day 1)	nab-paclitaxel 100 mg/m² (days 1/8/15) + carboplatin AUC 5–6 (day 1)	q3w, up to 35 cycles	PFS, OS, ORR

ORR, overall response rate; PFS, progression- free survival; OS, overall survival; pCR, pathologic complete response; MPR, major pathologic response; EFS, event-free survival; DFS, Disease-free survival; CG, control group; EG, experimental group; SCC, Squamous cell carcinoma; RR, rate ratio; HR, hazard ratio; 95% CI, 95% confidence interval.

### 2.4 Quality assessment

The Cochrane Collaboration tool was used to check the quality of each included study. We evaluated the risk of bias using Cochrane Collaboration Review Manager software (RevManVersion 5.3, Oxford, UK) (15). Each evaluated component was systematically assessed for potential bias, with classifications assigned to categories with low risk, high risk, or uncertain risk. In instances where discrepancies emerged during data extraction or quality appraisal, resolution was achieved through iterative group deliberation until unanimous consensus was attained.

### 2.5 Statistical analysis

The meta-analytic synthesis was performed using Cochrane Collaboration Review Manager software (RevManVersion 5.3, Oxford, UK). For each outcome, the appropriate effect measure was selected based on the type of data:

1. Time-to-event outcomes: Hazard ratios (HRs) with 95% confidence intervals (CIs) were used for OS, PFS, EFS and DFS.
2. Dichotomous outcomes: Odds ratios (ORs) with 95% CIs were used for ORR, pCR, MPR and the incidence of grade ≥3 adverse events (AEs), as these outcomes represent binary data.

A random-effects model was applied when significant heterogeneity was detected (*I^2^* >50% or *p*-value <0.05), a p-value threshold of *p* < 0.10 was used to define statistical heterogeneity due to the limited number of studies (k < 10) and to mitigate the risk of Type II error. Otherwise, a fixed-effects model was employed. Statistical significance was defined as a two-tailed *p*-value <0.05 or *p*-value <0.1 (conservative threshold for small study numbers).

## 3 Results

### 3.1 Literature screening

**Figure 1:**
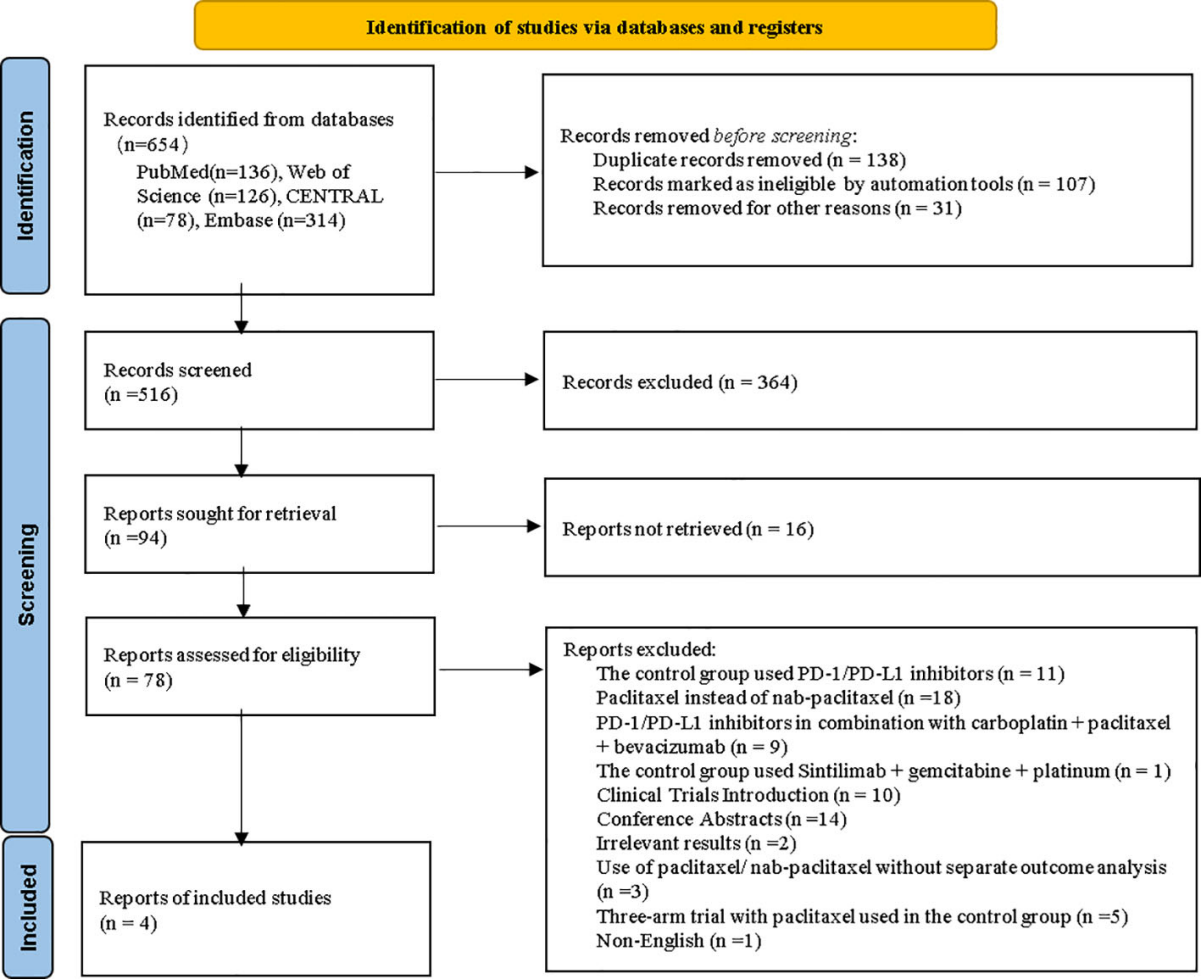
Flow chart of study selection according to PRISMA guidelines. RCT, Randomized controlled trial.

A systematic search across four academic databases identified 654 publications investigating PD-1/PD-L1 inhibitors plus nab-paclitaxel and platinum drugs for patients with non-small cell carcinoma. Following the removal of 138 redundant entries, the remaining 516 records underwent rigorous screening. This involved sequential evaluation of titles, abstracts, and full texts using predefined eligibility criteria, resulting in the elimination of 78 non-conforming studies. Ultimately, four eligible articles (10, 17–21) met inclusion requirements for this review. The comprehensive selection workflow, including exclusion rationales at each stage, was visualized in **Figure 1**.

### 3.2 Summary of study and participant characteristics

The meta-analysis incorporated data from 1998 participants across four eligible investigational studies that satisfied our inclusion criteria. Patient demographics were detailed in **Table 1**. We also outlined the pharmacotherapeutic approaches referenced in the reviewed publications in **Table 2**. A total of four clinical trials that met the defined eligibility criteria were incorporated into the analysis. Of the entire study, 1208 subjects were administered PD-1/PD-L1 inhibitors in conjunction with nab-paclitaxel and platinum-based agents (experimental group), while 790 subjects received nab-paclitaxel and platinum-based agents alone (control group).

The baseline demographic and clinical characteristics of the patient populations from the four included RCTs are comprehensively detailed in **Supplementary Table S1**. Overall, the trials encompassed distinct patient groups. The IMPower131 (Jotte et al.) and IMPower130 (West et al.) trials were multinational, enrolling patients with advanced non-squamous NSCLC. In contrast, the ASTRUM-004 (Zhou et al.) trial and the study by Lei et al. were conducted in China. ASTRUM-004 exclusively enrolled patients with squamous NSCLC, while the population in the Lei et al. study comprised treatment-naïve patients with resectable stage IIIA/IIIB disease.

The median age was consistent across all trials, ranging from 61 to 65 years. The majority of patients were male (59.0%–93.3%), had an ECOG performance status of 1 (57.9%–85.5%), and had a history of current or former smoking (72.1%–92.9%). The distribution of critical prognostic factors, such as the presence of liver metastases and brain metastases, was generally balanced between the intervention and control groups within each individual trial, supporting the validity of the subsequent comparative analyses.

### 3.3 Methodological quality

**Figure 2:**
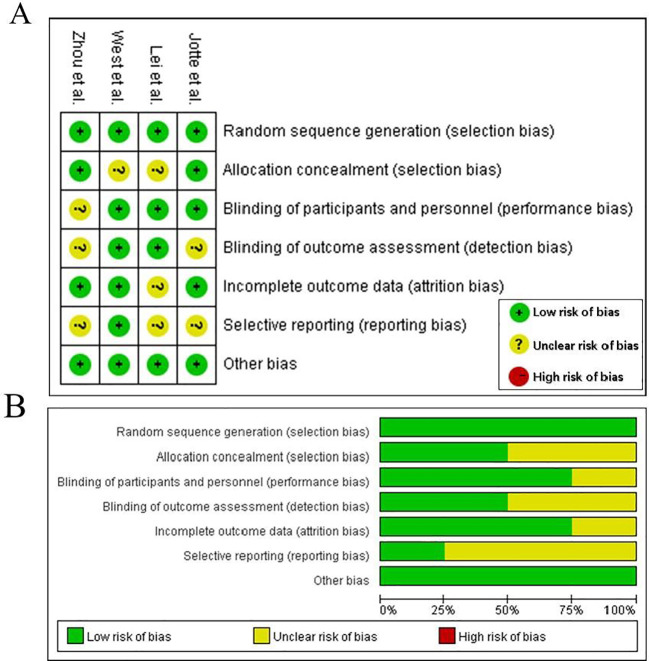
Results for assessment of the risk of bias. **(A)** Judgments of authors about each risk of bias item for each included study, **(B)** about each risk of bias item presented as percentages across all included studies.

The Cochrane risk-of-bias assessment highlighted critical variations in methodological rigor across the included studies (**Figures 2A, B**). All four included studies demonstrated a low risk of bias in random sequence generation. Incomplete outcome data and selective reporting were rated as low risk across all studies. However, allocation concealment was judged as unclear in two studies, and blinding of participants/personnel as well as outcome assessment showed a high risk of bias in half of the studies.

### 3.4 Efficacy analysis

#### 3.4.1 Objective response rate

**Figure 3:**
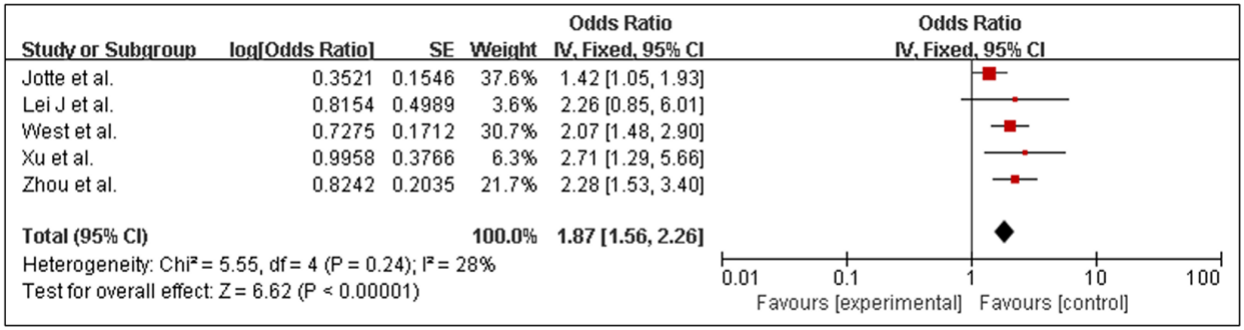
Forest plots of the meta-analysis of the effect of PD-1/PD-L1 inhibitors combined with nab-paclitaxel and platinum chemotherapy *vs*. nab-paclitaxel and platinum chemotherapy on ORR.

The meta-analysis incorporated four studies (10, 17, 18, 20) evaluating the ORR of the experimental group versus the control group. The pooled odds ratio for ORR between the experimental and control groups was 1.81(95% CI: 1.49–2.20, *p* < 0.001), favoring the PD-1/PD-L1 inhibitor combination therapy. No significant heterogeneity was observed across studies (*I²* = 54%, *p* = 0.11), supporting the use of a fixed-effects model. These results suggest a clinically meaningful improvement in ORR with the addition of PD-1/PD-L1 inhibitors to standard chemotherapy regimens, as illustrated in the forest plot (**Figure 3**). Among the included studies, two (18, 20) reported ORR outcomes in subgroups stratified by PD-L1 expression levels. Due to fundamental differences in PD-L1 assessment assays (TPS or TC/IC) and their associated scoring criteria, a meta-analytic pooled estimate was not calculated. In subgroup analyses based on PD-L1 expression, the objective response rate (ORR) was higher in the experimental arm across categories (**Table 3**).

**Table 3:** Objective response rate by PD-L1 subgroup of the included studies.

Study (Intervention)	PD-L1 Assay	PD-L1 Subgroup definition	ORR, %		OR (95% CI)
			EG	CG	
Jotte et al.	SP142 (Ventana) (Assesses Tumor Cells [TC] and Immune Cells [IC])	High (TC3 or IC3)	61.7	31.8	not provided
		Low (TC1/2 or IC1/2)	52.6	43.5	
		Negative (TC0 and IC0)	43.8	41.5	
Zhou et al. (18)	22C3 pharmDx (Agilent) (Tumor Proportion Score)	TPS ≥50%	66.3	43.4	2.57 (1.30 - 5.09)
		1% ≤ TPS <50%	52.1	29.3	2.60 (1.33 - 5.11)
		TPS <1%	62.2	47.1	1.92 (1.04 - 3.55)

#### 3.4.2 Pathologic response rates

Due to differences in clinical setting and endpoint definitions, the pathologic response outcomes (pCR and MPR) from the neoadjuvant TD-FOREKNOW trial by Lei et al. was not pooled with the radiographic ORR data from studies in advanced disease. In this trial, the camrelizumab-chemotherapy group demonstrated significantly higher pCR (32.6% vs. 8.9%; OR: 4.95, *p* = 0.008) and MPR (65.1% vs. 15.6%; OR: 10.13, *p* <.001) rates compared to chemotherapy alone, providing unique evidence of efficacy in the resectable setting.

#### 3.4.3 Progression-free survival

**Figure 4:**
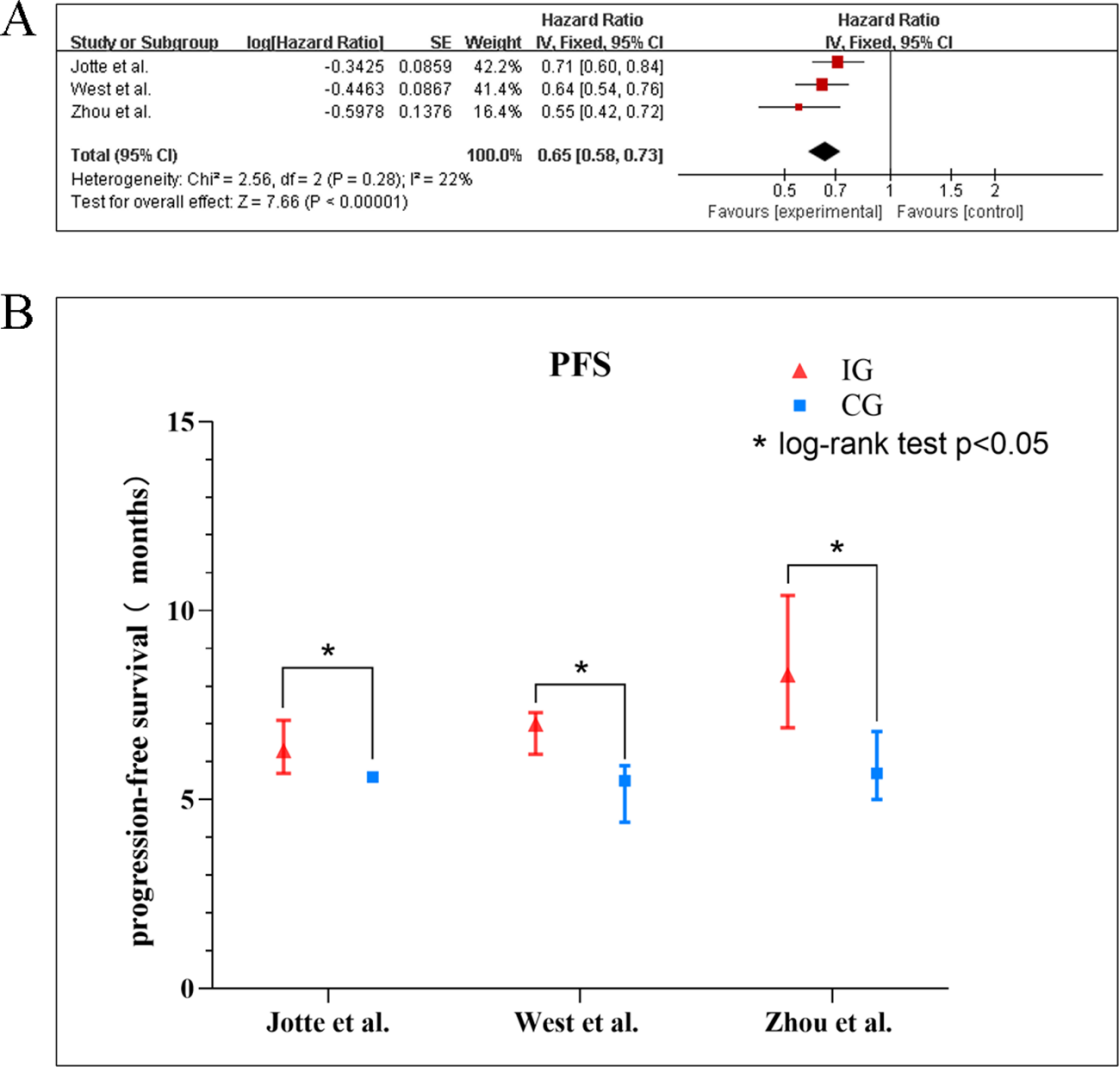
**(A)** Forest plots of the meta-analysis of the effect of PD-1/PD-L1 inhibitors combined with nab-paclitaxel and platinum chemotherapy *vs*. nab-paclitaxel and platinum chemotherapy on PFS. **(B)** Mean median PFS time of included studies. EG, control group; CG, experimental group; “*” represents log-rank test p < 0.05.

Based on the pooled hazard ratio in PFS of three studies (10, 18, 20), the meta-analysis revealed no significant heterogeneity among the included trials (*I^2^* = 22%, *p* = 0.28), supporting the use of a fixed-effects model. As illustrated in **Figure 4A**, the combined results demonstrated a statistically significant improvement in PFS favoring the experimental intervention over the control group (HR: 0.65, 95% CI: 0.58–0.73, *p* < 0.001). Individual study estimates consistently favored the experimental arm, with hazard ratios ranging from 0.55 (18) to 0.71 (20). The subgroup analysis (**Table 4**, **Supplementary figure 1**) for PFS revealed consistent and statistically significant benefits favoring PD-1/PD-L1 inhibitors combined with chemotherapy across all subgroups (pooled HR range: 0.48–0.77, all *p* < 0.05). Each subgroup derived a significant PFS benefit from the combination therapy (all *p* < 0.001). The point estimates suggested a trend toward greater benefit in females (HR = 0.55) than in males (HR = 0.64) and in patients aged ≥65 years (HR = 0.61) than in those <65 years (HR = 0.66). However, the tests for subgroup differences showed that these apparent variations were not statistically significant (for gender: *p* = 0.14; for age: *p* = 0.55). Similar nonsignificant differences in subgroup were also observed for ECOG PS 0/1 (*p* = 0.67) and liver metastases (*p* = 0.92). Notably, PD-L1 expression levels demonstrated significant subgroup variation (*I²* = 70.1%, *p* = 0.04), reinforcing its predictive value for immunotherapy response. In the study by Zhou et al. using TPS-based PD-L1 assessment, all subgroups (TPS <1%, 1–50%, and ≥50%) demonstrated significant PFS benefits with combination therapy (TPS <1%, HR = 0.46, 95% CI: 0.31–0.67; TPS 1–50%, HR = 0.71, 95% CI: 0.47–1.09; TPS ≥50%, HR = 0.44, 95% CI: 0.28–0.68). In three of the four trials analyzed, the median PFS was statistically significant (*p* < 0.05) for the experimental and control groups. Median PFS ranged from 6.3 to 8.3 months (mean7.2 months) in the experimental group and 5.5 to 5.7 months (mean 5.6months) in the control group (**Figure 4B**).

**Table 4:** Subgroup analysis of progression-free survival.

Subgroup of PFS	Number of studies	95%CI	*I* ^2^	P-Heterogeneity	P-value
Gender					
Male	3	0.64 (0.57, 0.72)	61%	0.08	p<0.001
Female	3	0.55 (0.48, 0.64)	3%	0.36	p<0.001
Overall effect		0.60 (0.55, 0.66)	47%	0.09	p<0.001
Subgroup differences			53%		0.14
Age					
<65 years	3	0.66 (0.57, 0.76)	50%	0.14	p<0.001
≥65 years	2	0.61 (0.49, 0.75)	0%	0.44	p<0.001
Overall effect		0.64 (0.57, 0.73)	18%	0.3	p<0.001
Subgroup differences			0%		0.55
ECOG PS					
0	3	0.62 (0.51, 0.76)	0%	0.63	p<0.001
1	3	0.66 (0.57, 0.75)	0%	0.4	p<0.001
Overall effect		0.64 (0.58, 0.72)	0%	0.71	p<0.001
Subgroup differences			0%		0.67
Liver metastases					
YES	3	0.70 (0.52, 0.94)	49%	0.14	p<0.001
NO	3	0.65 (0.49, 0.88)	70%	0.03	p<0.001
Overall effect		0.66 (0.55, 0.79)	53%	0.06	p<0.001
Subgroup differences			0%		0.92
PD-L1 Expression Level					
High	2	0.48 (0.35, 0.66)	0%	0.65	p<0.001
Low	2	0.66 (0.53, 0.83)	0%	0.55	P<0.001
Negative	2	0.77 (0.65, 0.91)	0%	0.50	P=0.002
Overall effect		0.68(0.60, 0.77)	35%	0.17	p<0.001
Subgroup differences			70.1%		0.04

95% CI, 95% confidence interval.

#### 3.4.4 Overall survival

**Figure 5:**
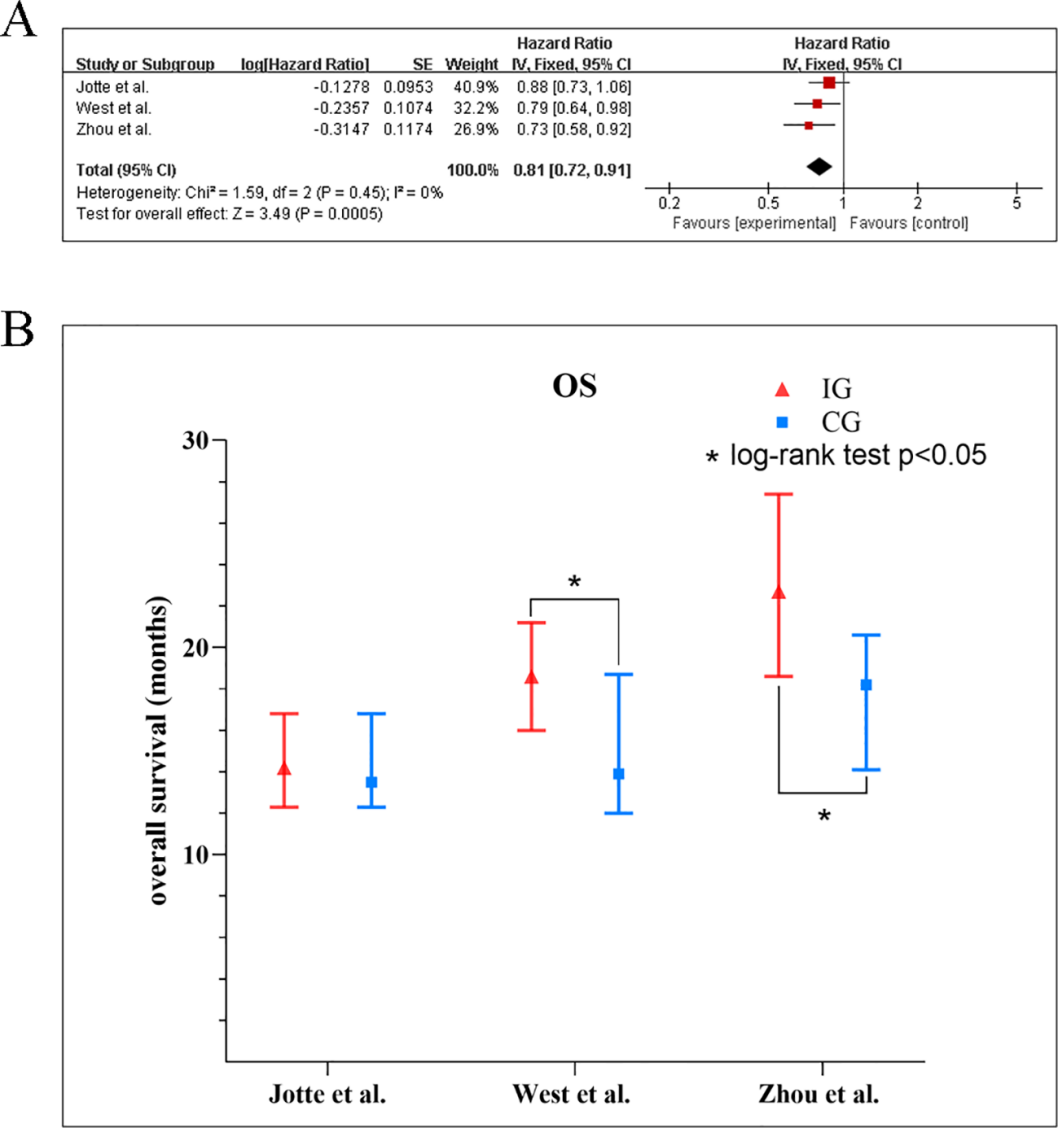
**(A)** Forest plots of the meta-analysis of the effect of PD-1/PD-L1 inhibitors combined with nab-paclitaxel and platinum chemotherapy *vs*. nab-paclitaxel and platinum chemotherapy on OS. **(B)** Mean median OS time of included studies. EG, control group; CG, experimental group; “*” represents log-rank test p < 0.05.

The encompassed three (10, 18, 20) studies that supplied the data of OS indicated low heterogeneity across the trials (*I^2^* = 0%, *p* = 0.64), justifying the application of a fixed-effect model. The synthesized outcomes, as depicted in the **Figure 5A**, revealed a statistically meaningful advantage in OS for the experimental group compared to the control group (HR: 0.81, 95% CI: 0.72–0.91; *p* < 0.001). Across individual investigations, hazard ratios uniformly supported the experimental intervention, varying from 0.73 (21) to 0.88 (17, 18). The subgroup analysis for OS (**Table 5**, **Supplementary figure 2**) showed a consistent survival benefit with the combination therapy in most predefined subgroups, including gender, age, ECOG PS, and liver metastasis status, with no significant subgroup differences (all p > 0.05). However, substantial heterogeneity was observed within PD-L1-based subgroups (*I²* = 41% overall). Significant OS improvement was identified in the PD-L1 high-expression subgroup (HR: 0.64, 95% CI: 0.45–0.91), whereas no significant benefit was observed in the low-expression subgroup (HR: 0.95, 95% CI: 0.75–1.21). In two of three studies, the experimental group obtained statistically significant results in relation to a longer mean OS than the control group. In the studies by West et al. (18.6 vs. 13.9 months, *p* = 0.033) (10) and Zhou et al. (22.7 vs. 18.2 months, *p* = 0.01) (18), the experimental group showed a significant increase in OS, whereas in the study by Jotte et al., a non-significant trend was observed (**Figure 5B**).

**Table 5:** Subgroup analysis of overall survival.

Subgroup of OS	Number of studies	95%CI	*I* ^2^	P-Heterogeneity	P-value
Gender					
Male	2	0.70 (0.59, 0.83)	0%	0.74	p<0.001
Female	2	0.79 (0.63, 0.98)	29%	0.23	p=0.03
Overall effect		0.73 (0.64, 0.83)	0%	0.53	p<0.001
Subgroup differences			0%		0.41
Age					
<65 years	2	0.85 (0.69, 1.04)	0%	0.57	p=0.10
≥65 years	1	0.78 (0.58, 1.05)	–	–	p=0.10
Overall effect		0.82 (0.70, 0.97)	0%	0.77	p=0.02
Subgroup differences			0%		0.66
ECOG PS					
0	2	0.74 (0.59, 0.93)	0%	0.35	p=0.009
1	2	0.72 (0.61, 0.85)	0%	0.59	p<0.001
Overall effect		0.73 (0.64, 0.83)	0%	0.75	p<0.001
Subgroup differences			0%		0.87
Liver metastases					
YES	2	0.74 (0.61, 0.91)	0%	0.81	p=0.004
NO	2	0.72 (0.60, 0.86)	59%	0.12	p<0.001
Overall effect		0.73 (0.64, 0.83)	0%	0.47	p<0.001
Subgroup differences			0%		0.81
Expression Level					
High	2	0.64 (0.45, 0.91)	58%	0.12	p=0.11
Low	2	0.95 (0.75, 1.21)	62%	0.11	p=0.62
Negative	2	0.84 (0.69, 1.02)	0%	0.72	p=0.08
Overall effect		0.84 (0.73, 0.96)	41%	0.13	p=0.04
Subgroup differences			40%		0.19

95% CI, 95% confidence interval; ECOG PS, Eastern Cooperative Oncology Group performance status.

#### 3.4.5 Event-free and disease-free survival

The EFS and DFS outcomes reported by Lei et al. (17) were analyzed separately as they are distinct endpoints specific to perioperative trials and are not directly comparable to the PFS/OS from studies in metastatic disease. With a median follow-up of 14.1 months, the median EFS and DFS were not reached in either group. The HR was 0.52 (95% CI: 0.21–1.29) for EFS and 0.54 (95% CI: 0.19–1.54) for DFS, favoring the camrelizumab combination. These results, while immature and not part of the meta-analysis, contribute preliminary evidence suggesting a potential survival benefit for camrelizumab plus nab-paclitaxel and platinum chemotherapy in this patient population.

### 3.5 Safety analysis

The safety profile of PD-1/PD-L1 inhibitors combined with nab-paclitaxel and platinum-based regimens was systematically analyzed using data from four studies (10, 10, 17–21), with AEs categorized into five domains: hematologic, gastrointestinal, neurologic, immune-related, and other reactions. Key AEs grade ≥ 3 were categorized by system and summarized with pooled risk ratios (RR) and 95% confidence intervals (CIs) (**Table 6**). A forest plot of Key AEs grade ≥ 3 was shown in **Supplementary Figure 3**. The overall incidence of treatment-associated adverse events (AEs) was significantly higher in the experimental group (RR 1.19, 95% CI: 1.12–1.27). Among hematologic AEs, thrombocytopenia was significantly increased in the experimental group (RR 1.83, 95% CI: 1.14–2.94), while no significant differences were observed for anemia (RR 0.90, 95% CI: 0.43–1.91), neutropenia (RR 0.62, 95% CI: 0.26–1.49), leukopenia (RR 0.96, 95% CI: 0.29–3.15), or decreased neutrophil count (RR 1.05, 95% CI: 0.78–1.41). Immune-related AEs emerged as the most pronounced safety concern, with a 2.49-fold higher risk in the immunotherapy group (RR: 2.49, 95% CI: 1.71–3.63). In contrast, gastrointestinal AEs such as nausea (RR 1.57, 95% CI: 0.61–4.03; 2 studies), diarrhea (RR 0.97, 95% CI: 0.54–1.75; 2 studies), and vomiting (RR 1.88, 95% CI: 0.63–5.65; 3 studies) did not significantly differ between treatment arms. Similarly, neurologic AEs including fatigue (RR 1.05, 95% CI: 0.63–1.75; 3 studies) and asthenia (RR 1.33, 95% CI: 0.66–2.70; 3 studies) also showed no significant differences. Other AEs, including hypomagnesemia and decreased platelet count, also showed no significant disparities. Notably, alopecia demonstrated an elevated but statistically unstable risk (RR: 3.14, 95% CI: 0.13–74.95), likely due to rare occurrences (1/377 vs. 0/379).

**Table 6:** The meta-analysis result of the adverse events in comparative studies.

Adverse events		N	EG	EG	Sample size	RR (95% CI)
			events/total	events/total		
Treatment-associated adverse events		4	768/1208	439/790	1998	1.19 (1.12, 1.27)
Hematologic						
	Anemia	3	212/1165	135/745	1910	0.90 (0.43, 1.91)
	Neutropenia	3	194/1165	154/745	1910	0.62 (0.26, 1.49)
	Thrombocytopenia	3	64/1165	19/745	1910	1.83 (1.14, 2.94)
	Leukopenia	4	48/1208	29/790	1998	0.96 (0.29, 3.15)
	Neutrophil count decreased	4	120/1208	64/790	1998	1.05 (0.78, 1.41)
Gastrointestinal						
	Nausea	2	16/807	6/566	1373	1.57 (0.61, 4.03)
	Diarrhoea	2	29/807	18/566	1373	0.97 (0.54, 1.75)
	Vomiting	3	13/1165	3/745	1910	1.88 (0.63, 5.65)
Neurologic						
	Fatigue	3	39/1165	22/745	1910	1.05 (0.63, 1.75)
	Asthenia	3	22/1165	12/745	1910	1.33 (0.66, 2.70)
Other adverse reactions						
	Alopecia	2	1/377	0/379	756	3.14 (0.13, 74.95)
	Hypomagnesaemia	2	5/807	5/566	1373	0.58 (0.17, 2.02)
	Platelet count decreased	1	37/473	14/232	705	1.30 (0.72, 2.35)
immune-related adverse event		4	118/1208	30/790	1998	2.49 (1.71, 3.63)

N, number of studies included; CG, control group; EG, experimental group; RR, rate ratio; 95% CI, 95% confidence interval.

### 3.6 Sensitivity analyses and publication bias

**Figure 6:**
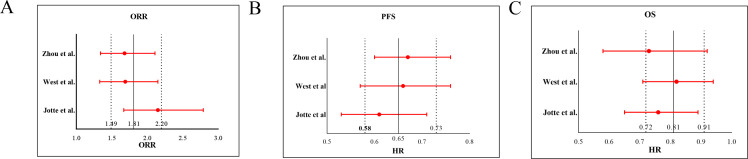
Sensitivity analysis of the **(A)** objective response rate (ORR), **(B)** progression-free survival (PFS), and **(C)** overall survival (OS).

**Figure 7:**
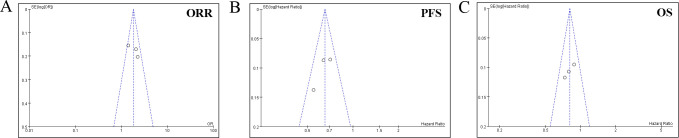
Funnel plot for the **(A)** objective response rate (ORR), **(B)** progression-free survival (PFS), and **(C)** overall survival (OS).

Sensitivity analysis employing a leave-one-out approach revealed stable pooled estimates for OS, PFS, and ORR, with no single study disproportionately influencing the results (**Figure 6**). Given the limited number of studies (k=4), formal statistical tests for funnel plot asymmetry (e.g., Egger’s test) were not performed, as they are known to be underpowered and potentially misleading in such cases. Instead, we applied a conservative, multi-faceted assessment: (1) Visual inspection of the funnel plot (**Figure 7**) was performed; (2) The fail-safe N method (*Nfs*​, Rosenthal’s approach) was calculated. The formula is *Nfs*=(∑*Z/*1.645)^2^−*k* (*Z*, the effect size test statistic for each study; *k*, the number of studies included in the analysis). The *Nfs*​ values for PFS, OS, and ORR were 38, 67 and 14, respectively, indicating that 38, 67 and 14 unpublished null studies would be needed to nullify the observed significant effect for PFS, OS, and ORR, which provides qualitative insight albeit with known limitations. While these methods cannot rule out publication bias, they allow for a more cautious interpretation.

## 4 Discussion

This meta-analysis systematically evaluated the efficacy and safety of PD-1/PD-L1 inhibitors combined with nab-paclitaxel and platinum-based chemotherapy in 1,998 patients with NSCLC across four RCTs, including both advanced and resectable disease settings. The pooled results demonstrated significant improvements in ORR, PFS and OS for the immunotherapy-containing regimen compared to chemotherapy alone. Additionally, while thrombocytopenia and immune-related AEs were more frequent in the experimental arm, other toxicities remained comparable between groups. These findings align with prior evidence supporting the integration of immune checkpoint inhibitors (ICIs) into first-line NSCLC treatment paradigms and extend their relevance to the perioperative setting (22).

The observed improvement in ORR (OR = 1.81) underscores the synergistic potential of combining ICIs with nab-paclitaxel-platinum chemotherapy. This enhanced antitumor activity may be attributed to nab-paclitaxel’s immunomodulatory effects, which are thought to create a more favorable tumor microenvironment for ICIs. Preclinical studies have demonstrated that nab-paclitaxel, more efficiently than solvent-based formulations, can promote the translocation of calreticulin to the cell surface, a key damage-associated molecular pattern (DAMP) signaling immunogenic cell death (ICD) (23). This, in turn, facilitates enhanced antigen uptake and presentation by dendritic cells, leading to a more robust priming of tumor-specific T-cell responses (24–26). Furthermore, clinical studies have observed a reduction in circulating immunosuppressive myeloid-derived suppressor cells (MDSCs) following nab-paclitaxel treatment, providing a plausible biological mechanism for the improved efficacy seen in our analysis (27). This multifaceted immunomodulation potentially synergizes with the mechanism of action of PD-1/PD-L1 inhibitors, which function by reversing T-cell exhaustion (24, 28).

All treatment regimens evaluated in the included trials were administered as first-line therapies for patients with advanced or resectable NSCLC. The PD-1/PD-L1 inhibitors were given concurrently with chemotherapy, not as a sequential or forced subsequent option. This design is pivotal, as concurrent administration maximizes the potential for synergistic immuno-chemotherapeutic effects by leveraging chemotherapy-induced immunogenic cell death to enhance the efficacy of simultaneous immune checkpoint blockade (1, 23, 28).

Notably, the efficacy of this combination extends beyond the metastatic setting. As demonstrated in the TD-FOREKNOW trial by Lei et al. (17), which exclusively enrolled patients with resectable stage IIIA/IIIB disease, the neoadjuvant use of camrelizumab plus nab-paclitaxel/platinum resulted in dramatically higher pathologic complete response (pCR) and major pathologic response (MPR) rates compared to chemotherapy alone (32.6% vs. 8.9% and 65.1% vs. 15.6%, respectively). This aligns with the findings from the landmark CheckMate 816 trial of neoadjuvant nivolumab plus chemotherapy (29), confirming that the synergy between immunotherapy and chemotherapy is potent in the perioperative setting, potentially leading to improved long-term survival outcomes by eradicating micrometastatic disease. The safety profile in this resectable population was manageable and did not preclude successful surgery, which is a critical consideration for neoadjuvant strategies (30).

The PFS benefit (HR = 0.65, 95% CI: 0.58–0.72) and OS advantage (HR = 0.81, 95% CI: 0.72–0.91) in the advanced disease trials further validate the durability of this combination. These findings are consistent with the established survival benefits of ICI-chemotherapy combinations seen in broader populations (3, 21). Our results align with a recent network meta-analysis by Zhao et al. (31), which compared various ICI-chemotherapy regimens and found that nab-paclitaxel-based combinations ranked highly for both PFS and OS in squamous NSCLC. Notably, subgroup analyses revealed stronger PFS benefits in patients with high PD-L1 expression, aligning with its established role as a predictive biomarker. However, the lack of a significant OS benefit in PD-L1-negative subgroups, combined with the considerable response observed in some of these patients, underscores the limitations of PD-L1 as a standalone biomarker and highlights the critical need for a more comprehensive, multi-factorial approach to patient selection (32).

While our analysis was necessarily focused on PD-L1—as it was the primary biomarker reported in the included trials—the field is rapidly evolving to encompass other promising biomarkers. Tumor mutational burden (TMB) serves as a powerful complement, predicting response to immunotherapy independent of PD-L1 status by reflecting neoantigen burden (28, 33, 34). Beyond genomic features, the tumor immune microenvironment (TME) provides crucial contextual information; the density and location of CD8+ tumor-infiltrating lymphocytes (TILs) are strong positive predictors of response (35), whereas upregulation of alternative immune checkpoints (LAG-3, TIM-3) can signify adaptive resistance mechanisms (36). Furthermore, systemic host factors, such as a derived neutrophil-to-lymphocyte ratio (dNLR), offer readily accessible proxies for an immunosuppressive state (37), and compelling evidence now links the composition of the gut microbiome to immunotherapy efficacy through modulation of host immunity (38). Future studies should integrate multifaceted biomarkers—including tumor-intrinsic, microenvironmental, and host-related factors—into unified models to optimally identify patients who will benefit most from this combination regimen.

In terms of safety, the increased risk of thrombocytopenia (RR = 1.83) and immune-related AEs (RR = 2.49) in the experimental group is consistent with prior meta-analyses of ICI-chemotherapy combinations (13, 39). However, the clinical implications of these findings extend beyond statistical significance and warrant careful consideration, as they directly impact treatment feasibility and adoption in real-world settings. Grade ≥3 thrombocytopenia can necessitate treatment delays, dose reductions, or even discontinuation, potentially compromising treatment intensity and efficacy. It also requires vigilant monitoring, interventions such as platelet transfusions, and increases the risk of bleeding events, thereby adding complexity and cost to patient management (40). The near 2.5-fold increased risk of immune-related adverse events (irAEs) presents an even greater challenge. irAEs often demand prompt recognition, specialist involvement, and prolonged courses of corticosteroids or other immunosuppressants, which are associated with their own morbidities and can diminish quality of life (41). The management burden of these toxicities significantly influences the real-world adoption of this regimen. In clinical practice, the successful and safe implementation of this combination is contingent upon institutional preparedness, including established management protocols and multidisciplinary support—resources that may not be uniformly available across all treatment centers (42). Our trial-based findings are further contextualized by real-world pharmacovigilance data. Analyses of freely available databases like the FDA Adverse Event Reporting System (FAERS) provide insights into the “real-life” incidence and nature of these ADRs. For example, a large-scale FAERS analysis not only confirms the signal for immune-mediated thrombocytopenia but also reveals a spectrum of associated clinical manifestations, such as severe bleeding events, and identifies drug-specific risk differences (43). Moreover, such databases are invaluable for detecting rare but fatal toxicities (e.g., ICI-associated myocarditis) that may be under-represented in clinical trials due to their limited sample size (44).

Therefore, while the therapeutic efficacy of this combination regimen is compelling, clinicians must engage in shared decision-making with patients by carefully weighing its significant survival benefits against the increased risks of specific and manageable yet clinically consequential toxicities, such as thrombocytopenia and immune-related adverse events. This highlights the necessity of not only biomarker-driven patient selection but also proactive toxicity monitoring and management protocols. It is important to note, however, that the overall safety profile features favorable aspects: the incidence of severe neutropenia did not differ significantly from that in the control group, and the low rate of severe neurotoxicity confirms the preserved safety advantage of nab-paclitaxel over solvent-based paclitaxel (7, 16, 45).

When considering the broader landscape of immune-combination therapies, our findings invite comparison with other established first-line regimens. The significant survival benefit observed here is comparable to that achieved with pembrolizumab plus pemetrexed/platinum in patients with non-squamous NSCLC in KEYNOTE-189 trail (10). This suggests that nab-paclitaxel-based triplets represent a potent alternative backbone for immunotherapy in NSCLC. Furthermore, the efficacy in squamous NSCLC, as demonstrated in the ASTRUM-004 trial, fills an important niche, as treatment options for this subtype have historically been more limited compared to non-squamous carcinomas (11) The consistency of benefit across different PD-1/PD-L1 inhibitors (atezolizumab, serplulimab, camrelizumab) supports a class-effect phenomenon for this combination strategy, reinforcing the robustness of this therapeutic approach (4, 6). The potential universality and promotion of this combination regimen may extend beyond the current scope. The rationale of combining taxane-based chemotherapy with immunotherapy could be applicable to other solid malignancies. For instance, similar regimens are being explored in aggressive cancers like triple-negative breast cancer (38). Future research should focus on head-to-head comparisons between nab-paclitaxel and other chemotherapy backbones (e.g., pemetrexed, paclitaxel) in combination with the same ICI to definitively establish superiority, if any.

Our study has several limitations that must be acknowledged. First and foremost, the clinical and methodological heterogeneity across the included trials necessitates a nuanced interpretation of our pooled results. The analyses encompassed a spectrum of NSCLC disease stages (from resectable to advanced metastatic disease), histological subtypes (squamous and non-squamous), and different PD-1/PD-L1 inhibitors. While this diversity increases the generalizability of the regimen’s activity, it also introduces complexity. This heterogeneity informs a more cautious and scenario-specific application of our findings. The impressive improvement in pathological complete response (pCR) and major pathological response (MPR) rates, driven exclusively by the neoadjuvant trial (17), robustly supports the efficacy of this regimen for inducing tumor downstaging in operable patients. However, the overall survival (OS) and progression-free survival (PFS) benefits are primarily derived from the metastatic setting and should not be directly extrapolated to predict survival outcomes in the resectable population, where long-term follow-up data are still maturing. Furthermore, although our subgroup analyses and low statistical heterogeneity (*I²*) for OS and PFS suggest a consistent treatment effect, the limited number of trials may underpower the detection of true differences among subgroups. Similarly, the observed “class effect” across different PD-1/PD-L1 inhibitors is encouraging but does not preclude the possibility of subtle differences in efficacy or safety profiles among specific agents.

Additionally, our work has several other important limitations. First, the number of included studies was relatively small (k=4), which limited the statistical power of some subgroup analyses and increases the uncertainty around our estimates. This limitation is compounded by the fact that the review protocol was not prospectively registered in a public registry (e.g., PROSPERO). Although we adhered to a pre-defined internal protocol and followed PRISMA guidelines rigorously, the lack of public registration introduces a potential for selective reporting bias, as it may raise concerns about the pre-specification of outcomes and analyses (46). Second, as appropriately noted in methodological literature, the assessment of publication bias in a meta-analysis with few studies is inherently underpowered and unreliable (47). While we employed conservative methods, the potential for unpublished negative studies cannot be ruled out. Third, model selection in this meta-analysis was primarily based on quantitative measures of statistical heterogeneity (the *I²* statistic and the *p*-value from Cochrane’s Q test). Although this provides an objective framework, we acknowledge that future analyses should, as suggested by the Cochrane Handbook, more comprehensively integrate considerations of clinical and methodological heterogeneity to guide model choice. Finally, we could not perform a detailed analysis based on the specific PD-1/PD-L1 inhibitor used, as the mechanisms, while overlapping, may not be identical. Some research suggests differences in binding epitopes and Fc receptor interactions might lead to varying clinical effects.

## 5 Conclusion

This meta-analysis reinforces the clinical value of PD-1/PD-L1 inhibitors combined with nab-paclitaxel-platinum chemotherapy in NSCLC, albeit with increased risks of thrombocytopenia and immune-related toxicities. These results advocate for the adoption of this triplet regimen as a first-line therapeutic option, provided clinicians implement proactive AEs monitoring.

## Data Availability

The original contributions presented in the study are included in the article/**Supplementary Material**. Further inquiries can be directed to the corresponding author.
