# Supplementary material for: Synergistic efficacy and safety of PD-1/PD-L1 inhibitors combined with nab-paclitaxel and platinum chemotherapy in NSCLC: A systematic review and meta-analysis of randomized controlled trials
Source: Front Oncol. 2025 Dec 3;15:1649777. doi: 10.3389/fonc.2025.1649777 (PMC12708242; doi:10.3389/fonc.2025.1649777)
Supplement: Supplementary file 1 [file SupplementaryFile1.docx]

**Supplementary Material 1**

**Search Strategies**

**Date searched: January 24, 2025**

**Cochrane Central Register of Controlled Trials**

#1 MeSH descriptor: [Immune Checkpoint Inhibitors] explode all trees

#2 (immune checkpoint inhibitor OR Immune Checkpoint Inhibitors):ti,ab,kw

#3 MeSH descriptor: [Programmed Cell Death 1 Receptor] explode all trees

#4 (Programmed Cell Death 1 Receptor OR programmed cell death 1 protein OR pd 1 receptor):ti,ab,kw

#5 MeSH descriptor: [Nivolumab] explode all trees

#6 (Nivolumab OR opdivo OR BMS-936558 OR BMS936558 OR BMS-936558 OR ONO4538 OR ono 4538 OR MDX-1106 OR MDX1106 OR MDX-1106):ti,ab,kw

#7 (Toripalimab OR pembrolizumab OR Keytruda OR lambrolizumab OR MK-3475 OR MK3475 OR SCH-900475 OR Atezolizumab OR MPDL3280A OR MPDL-3280A OR RG7446 OR RG-7446 OR Tecentriq OR Sintilimab OR camrelizumab OR tislelizumab OR durvalumab):ti,ab,kw

#8 #1 OR #2 OR #3 OR #4 OR #5 OR #6 OR #7

#9 MeSH descriptor: [Albumin-Bound Paclitaxel] explode all trees

#10 (ABI007 OR ABI-007 OR ABI 007 OR Abraxane OR Albumin Bound Paclitaxel OR Paclitaxel, Albumin-Bound OR Protein-Bound Paclitaxel OR Paclitaxel, Protein-Bound OR Protein Bound Paclitaxel OR Nanoxel OR Paclical OR Nanoparticle Albumin-Bound Paclitaxel):ti,ab,kw

#11 #9 OR #10

#12 MeSH descriptor: [Carcinoma, Non-Small-Cell Lung] explode all trees

#13 (Non-Small-Cell Lung Carcinoma OR Lung Carcinomas, Non-Small-Cell OR Carcinoma, Non Small Cell Lung OR Carcinoma, Non-Small Cell Lung OR Non-Small-Cell Lung Carcinomas OR Lung Carcinoma, Non-Small-Cell OR Non Small Cell Lung Carcinoma OR Non-Small Cell Lung Cancer OR Carcinomas, Non-Small-Cell Lung OR Nonsmall Cell Lung Cancer OR Non-Small Cell Lung Carcinoma):ti,ab,kw

#14 #12 OR #13

#15 #8 AND #11 AND #14

**Pubmed**

#1 "Immune Checkpoint Inhibitors" [MeSH Terms]

#2 "immune checkpoint inhibitor"[Title/Abstract] OR "Immune Checkpoint Inhibitors"[Title/Abstract] OR "Programmed Cell Death 1 Receptor"[MeSH Terms] OR "Programmed Cell Death 1 Receptor"[Title/Abstract] OR "programmed cell death 1 protein"[Title/Abstract] OR "pd 1 receptor"[Title/Abstract] OR "Nivolumab"[MeSH Terms] OR "Nivolumab"[Title/Abstract] OR "opdivo"[Title/Abstract] OR "BMS-936558"[Title/Abstract] OR "BMS936558"[Title/Abstract] OR "BMS-936558"[Title/Abstract] OR "ONO4538"[Title/Abstract] OR "ono 4538"[Title/Abstract] OR "MDX-1106"[Title/Abstract] OR "MDX1106"[Title/Abstract] OR "MDX-1106"[Title/Abstract] OR "Toripalimab"[Supplementary Concept] OR "Toripalimab"[Title/Abstract] OR "pembrolizumab"[Supplementary Concept] OR "pembrolizumab"[Title/Abstract] OR "Keytruda"[Title/Abstract] OR "lambrolizumab"[Title/Abstract] OR "MK-3475"[Title/Abstract] OR "MK3475"[Title/Abstract] OR "MK-3475"[Title/Abstract] OR "SCH-900475"[Title/Abstract] OR "Atezolizumab"[Supplementary Concept] OR "Atezolizumab"[Title/Abstract] OR "MPDL3280A"[Title/Abstract] OR "MPDL-3280A"[Title/Abstract] OR "MPDL-3280A"[Title/Abstract] OR "RG7446"[Title/Abstract] OR "RG-7446"[Title/Abstract] OR "RG-7446"[Title/Abstract] OR "Tecentriq"[Title/Abstract] OR "Sintilimab"[Supplementary Concept] OR "Sintilimab"[Title/Abstract] OR "camrelizumab"[Supplementary Concept] OR "camrelizumab"[Title/Abstract] OR "tislelizumab"[Supplementary Concept] OR "tislelizumab"[Title/Abstract] OR "durvalumab"[Supplementary Concept] OR "durvalumab"[Title/Abstract]

#3 #1 OR #2

#4 "albumin bound paclitaxel"[MeSH Terms]

#5 "nab-paclitaxel"[Title/Abstract] OR "nab paclitaxel"[Title/Abstract] OR "nabpaclitaxel"[Title/Abstract] OR "albumin-bound paclitaxel"[Title/Abstract] OR "albumin bound paclitaxel"[Title/Abstract] OR "paclitaxel albumin-bound"[Title/Abstract] OR "paclitaxel albumin bound"[Title/Abstract] OR "protein-bound paclitaxel"[Title/Abstract] OR "paclitaxel protein-bound"[Title/Abstract] OR "Abraxane"[Title/Abstract] OR "ABI-007"[Title/Abstract] OR "ABI007"[Title/Abstract] OR "abi 007"[Title/Abstract] OR "Nanoxel"[Title/Abstract] OR "Paclical"[Title/Abstract] OR "nanoparticle albumin-bound paclitaxel"[Title/Abstract])

#6 #4 OR #5

#7 "Carcinoma, Non-Small-Cell Lung"[MeSH Terms] OR "Lung Neoplasms"[MeSH Terms]

#8 "non-small cell lung cancer"[Title/Abstract] OR "non small cell lung cancer"[Title/Abstract] OR "nonsmall cell lung cancer"[Title/Abstract] OR "non-small-cell lung carcinoma"[Title/Abstract] OR "NSCLC"[Title/Abstract] OR "non-small cell lung tumor"[Title/Abstract] OR "lung non-small cell carcinoma"[Title/Abstract] OR "non-small cell carcinoma of lung"[Title/Abstract] OR "non-small cell lung cancers"[Title/Abstract] OR "non-small cell lung malignancy"[Title/Abstract] OR "non-small cell lung carcino*"[Title/Abstract] OR "lung adenocarcinoma"[Title/Abstract] OR "lung squamous cell carcinoma"[Title/Abstract] OR "large cell lung carcinoma"[Title/Abstract])

#9 #7 OR #8

#10 #3 AND #6 AND #9

#11((((((randomized controlled trial [pt]) OR (controlled clinical trial [pt])) OR (randomized [Title/Abstract])) OR (placebo [Title/Abstract])) OR (clinical trials as topic [mesh: noexp])) OR (randomly [Title/Abstract])) OR (trial [ti])

#12 animals [mh] NOT humans [mh]

#13 #11 NOT #12

#14 #10 AND #13

**Web of science**

#1 TI=( Immune Checkpoint Inhibitors) OR AB=( Immune Checkpoint Inhibitors) OR TI=( Programmed Cell Death 1 Receptor) OR AB=( Immune Checkpoint Inhibitors OR Programmed Cell Death 1 Receptor OR programmed cell death 1 protein OR pd 1 receptor) OR TI=( Nivolumab OR opdivo OR BMS-936558 OR BMS936558 OR ONO4538 OR ono 4538 OR MDX-1106 OR MDX1106 OR Toripalimab OR pembrolizumab OR Keytruda OR lambrolizumab OR MK3475 OR MK-3475 OR SCH-900475 OR Atezolizumab OR MPDL-3280A OR MPDL-3280A OR RG7446 OR RG-7446 OR Tecentriq OR Sintilimab OR camrelizumab OR tislelizumab OR durvalumab) OR AB=( Nivolumab OR opdivo OR BMS-936558 OR BMS936558 OR ONO4538 OR ono 4538 OR MDX-1106 OR MDX1106 OR Toripalimab OR pembrolizumab OR Keytruda OR lambrolizumab OR MK3475 OR MK-3475 OR SCH-900475 OR Atezolizumab OR MPDL-3280A OR MPDL-3280A OR RG7446 OR RG-7446 OR Tecentriq OR Sintilimab OR camrelizumab OR tislelizumab OR durvalumab)

#2 TI=( Albumin-Bound Paclitaxel) OR AB=( Albumin-Bound Paclitaxel) OR TI=(ABI007 OR ABI-007 OR ABI 007 OR Abraxane OR Albumin Bound Paclitaxel OR Paclitaxel, Albumin-Bound OR Protein-Bound Paclitaxel OR Paclitaxel, Protein-Bound OR Protein Bound Paclitaxel OR Nanoxel OR Paclical OR Nanoparticle Albumin-Bound Paclitaxel) OR AB=(ABI007 OR ABI-007 OR ABI 007 OR Abraxane OR Albumin Bound Paclitaxel OR Paclitaxel, Albumin-Bound OR Protein-Bound Paclitaxel OR Paclitaxel, Protein-Bound OR Protein Bound Paclitaxel OR Nanoxel OR Paclical OR Nanoparticle Albumin-Bound Paclitaxel)

#3 TI =( Carcinoma, Non-Small-Cell Lung) OR AB=( Immune Checkpoint Inhibitors) OR AB=(Non-Small-Cell Lung Carcinoma OR Lung Carcinomas, Non-Small-Cell OR Carcinoma, Non Small Cell Lung OR Carcinoma, Non-Small Cell Lung OR Non-Small-Cell Lung Carcinomas OR Lung Carcinoma, Non-Small-Cell OR Non Small Cell Lung Carcinoma OR Non-Small Cell Lung Cancer OR Carcinomas, Non-Small-Cell Lung OR Nonsmall Cell Lung Cancer OR Non-Small Cell Lung Carcinoma)

#4 #1 AND #2 AND #3

**Database: Embase**

#1 'immune checkpoint inhibitor'/exp OR 'immune checkpoint inhibitor' OR 'Immune Checkpoint Inhibitors'

#2 'Programmed Cell Death 1 Receptor'/exp OR 'Programmed Cell Death 1 Receptor' OR 'Programmed Cell Death 1 Protein' OR 'PD-1 Receptor'

#3 'nivolumab'/exp OR 'opdivo' OR 'BMS-936558' OR 'BMS936558' OR 'BMS-936558' OR 'ONO4538' OR 'ono 4538' OR 'MDX-1106' OR 'MDX1106' OR 'MDX-1106'

#4 'SCH-900475' OR 'Atezolizumab' OR 'MPDL3280A' OR 'MPDL-3280A' OR 'RG7446' OR 'RG-7446' OR 'Tecentriq' OR 'Sintilimab' OR 'camrelizumab' OR 'tislelizumab' OR 'durvalumab'

#5 #1 OR #2 OR #3 OR #4

#6 'Albumin-Bound Paclitaxel'/exp OR 'ABI007' OR 'ABI-007' OR 'ABI 007' OR 'Abraxane' OR 'Albumin Bound Paclitaxel' OR 'Paclitaxel, Albumin-Bound' OR 'Protein-Bound Paclitaxel' OR 'Paclitaxel, Protein-Bound' OR 'Protein Bound Paclitaxel' OR 'Nanoxel' OR 'Paclical' OR 'Nanoparticle Albumin-Bound Paclitaxel'

#7 'Carcinoma, Non-Small-Cell Lung'/exp OR 'Non-Small-Cell Lung Carcinoma' OR 'Lung Carcinomas, Non-Small-Cell' OR 'Carcinoma, Non Small Cell Lung' OR 'Carcinoma, Non-Small Cell Lung' OR 'Non-Small-Cell Lung Carcinomas' OR 'Lung Carcinoma, Non-Small-Cell' OR 'Non Small Cell Lung Carcinoma' OR 'Non-Small Cell Lung Cancer' OR 'Carcinomas, Non-Small-Cell Lung' OR 'Nonsmall Cell Lung Cancer' OR 'Non-Small Cell Lung Carcinoma'

#8 'randomized controlled trial'/exp

#9 #5 AND #6 AND #7 AND #8

**Supplementary figure 1**

Forest plots of subgroup analysis of PFS in PD-L1 Expression Level between PD-1/PD-L1 Inhibitors Combined with Nab-Paclitaxel and Platinum Chemotherapy and Nab-Paclitaxel and Platinum Chemotherapy.

**
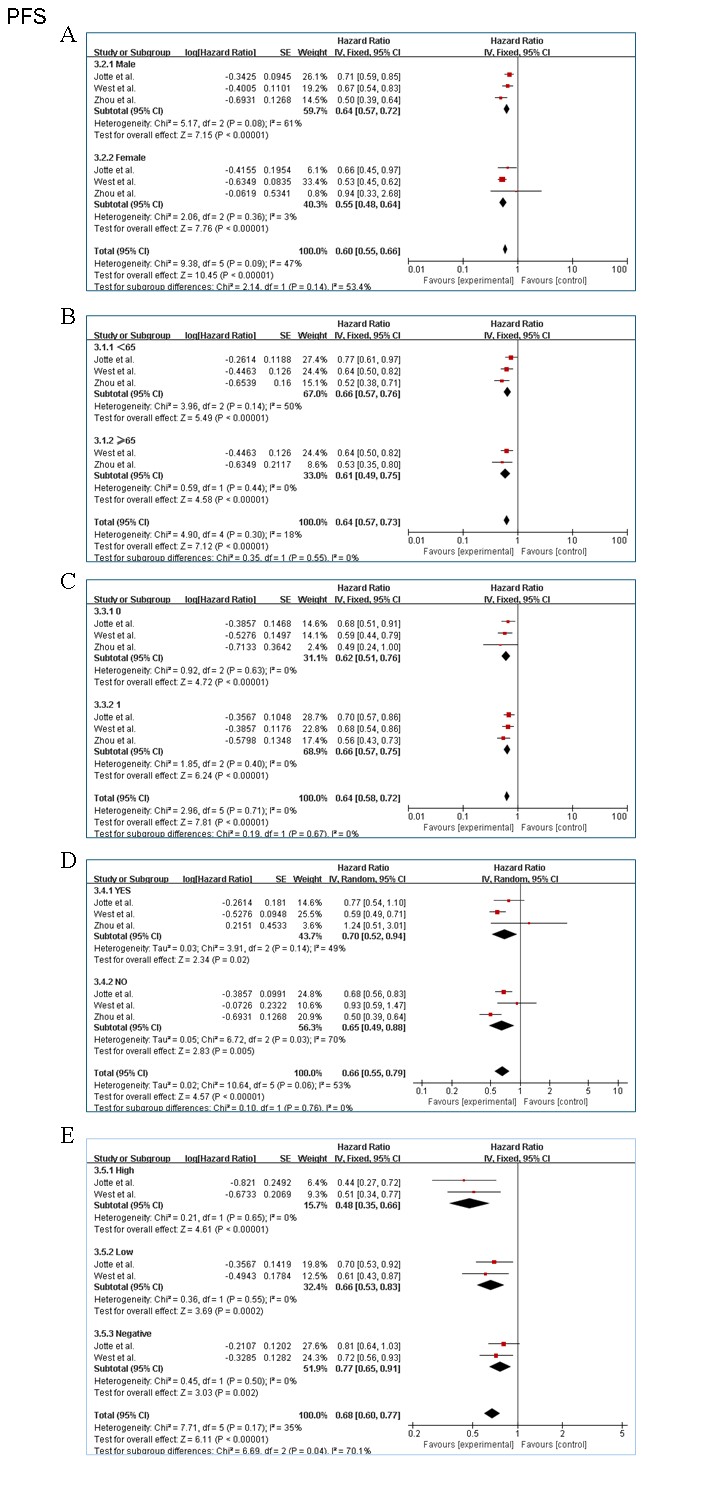
**

**Supplementary figure 2**

Forest plots of subgroup analysis of OS in PD-L1 Expression Level between PD-1/PD-L1 Inhibitors Combined with Nab-Paclitaxel and Platinum Chemotherapy and Nab-Paclitaxel and Platinum Chemotherapy.


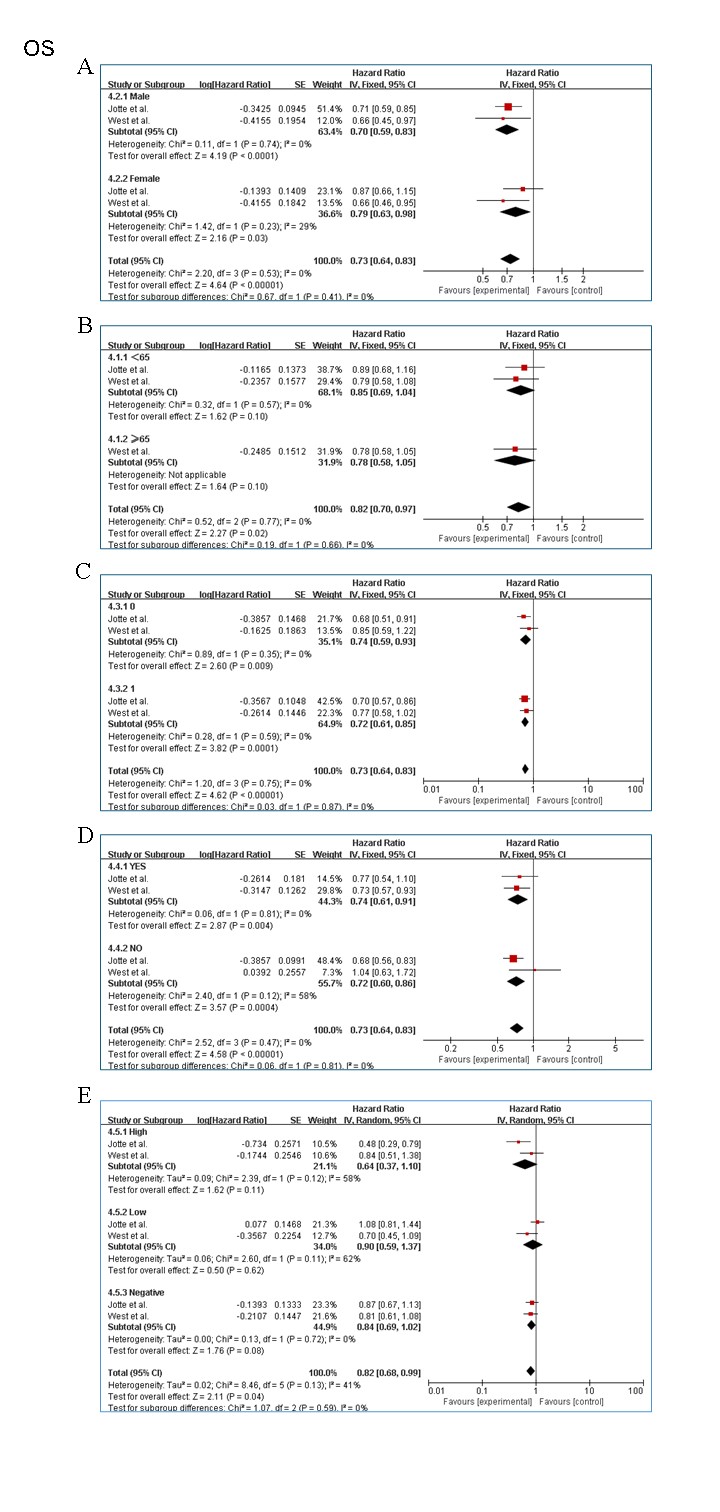


**Supplementary figure 3**

Forest plots of the meta-analysis of the effect of PD-1/PD-L1 Inhibitors Combined with Nab-Paclitaxel and Platinum Chemotherapy vs. Nab-Paclitaxel and Platinum Chemotherapy on Key adverse events grade ≥ 3.


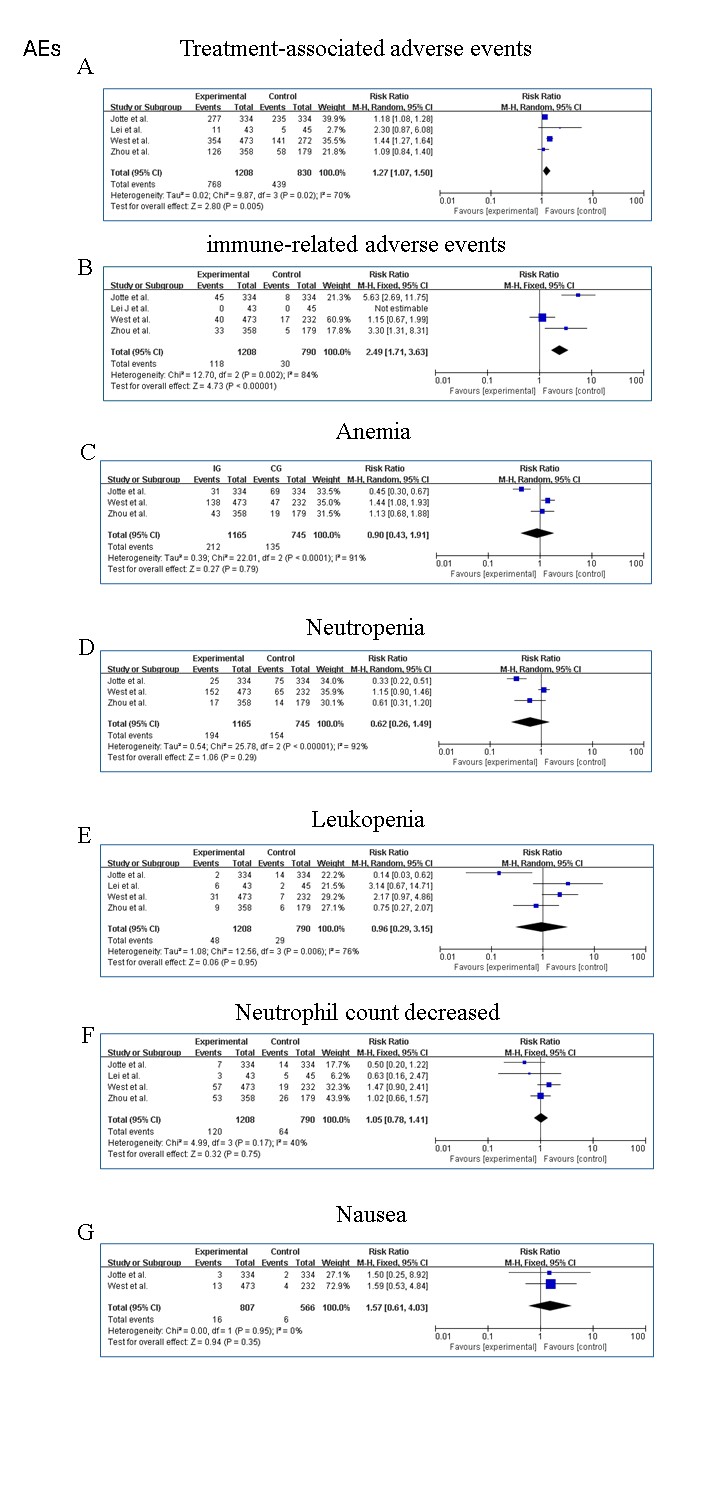

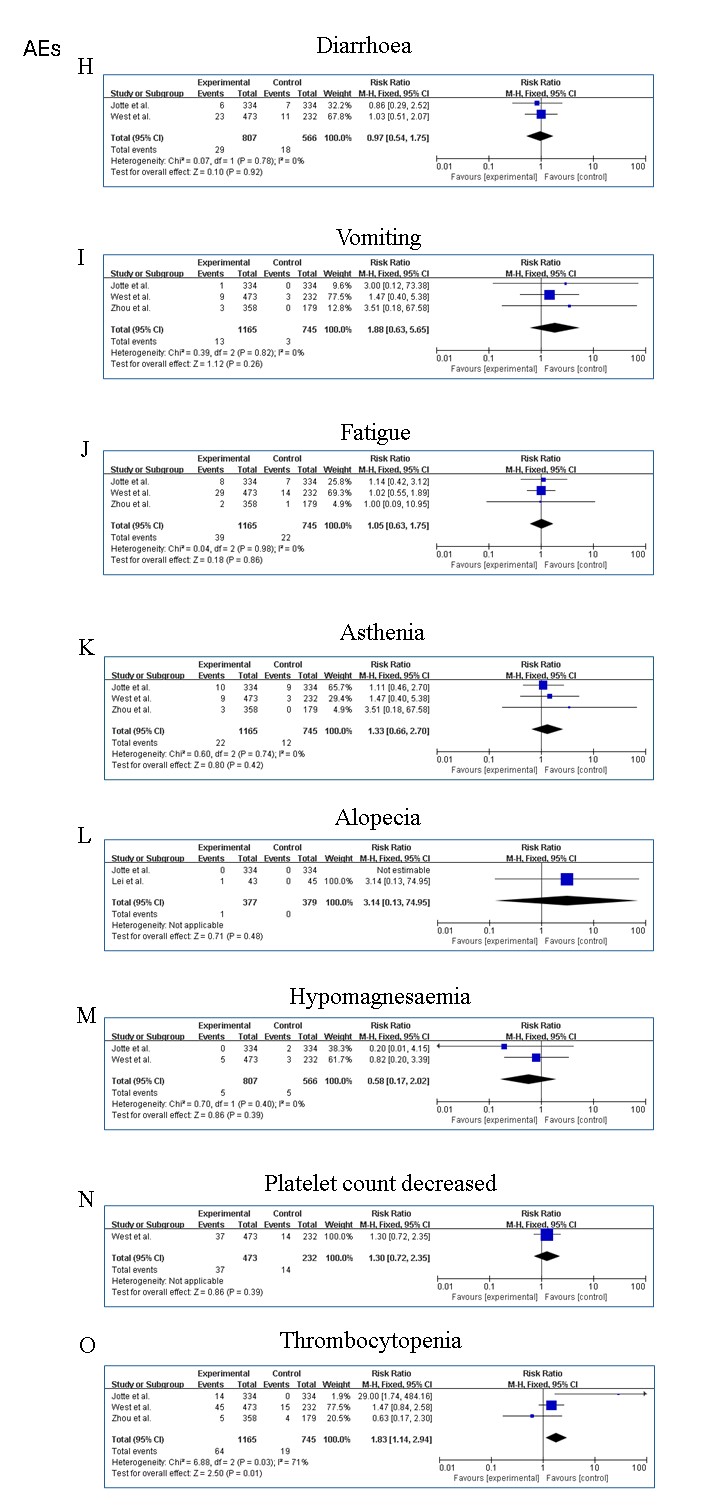


**Supplementary table S1**

**Table S1. Detailed Baseline Characteristics of Patients in the Included Studies**

| **Characteristic** | **Lei et al. (2023)** | | **Jotte et al. (2018) *IMPower131*** | | **West et al. (2019) *IMPower130*** | | **Zhou et al. (2024) ASTRUM-004** | |
| --- | --- | --- | --- | --- | --- | --- | --- | --- |
| Region | China | | Multinational | | Multinational | | China | |
| Treatment Groups | Cam+Chemo (n=43) | Chemo (n=45) | A+CnP (n=343) | CnP (n=340) | Atezo+Chemo (n=451) | Chemo (n=228) | Serp+Chemo (n=358) | Pla+Chemo (n=179) |
| Age, n (%) |  |  |  |  |  |  |  |  |
| <65 Years | - | - | 170 (49.6) | 156 (45.9) | 227 (50.3) | 114 (50.0) | - | - |
| 65-74 Years | - | - | 134 (39.1) | 145 (42.6) | 174 (38.6) | 84 (36.8) | - | - |
| 75-84 Years | - | - | 39 (11.4) | 38 (11.2) | 48 (10.6) | 29 (12.7) | - | - |
| ≥85 Years | - | - | 0 | 1 (0.3) | 2 (0.4) | 1 (0.4) | - | - |
| Sex, n (%) |  |  |  |  |  |  |  |  |
| Male | 34 (79.1) | 40 (88.9) | 280 (81.6) | 277 (81.5) | 266 (59.0) | 134 (58.8) | 321 (89.7) | 167 (93.3) |
| Female | 9 (20.9) | 5 (11.1) | 63 (18.4) | 63 (18.5) | 185 (41.0) | 94 (41.2) | 37 (10.3) | 12 (6.7) |
| Race, n (%) |  |  |  |  |  |  |  |  |
| Asian | - | - | 41 (12.0) | 37 (10.9) | 12 (2.7) | 3 (1.3) | 240 (67.0) | 119 (66.5) |
| White | - | - | 289 (84.3) | 290 (85.3) | 402 (89.1) | 210 (92.1) | 118 (33.0) | 60 (33.5) |
| Black | - | - | 4 (1.2) | 7 (2.1) | 17 (3.8) | 8 (3.5) | - | - |
| Other/Unknown | - | - | 9 (2.6) | 6 (1.8) | 20 (4.4) | 7 (3.1) | 0 | 0 |
| ECOG PS, n (%) |  |  |  |  |  |  |  |  |
| 0 | 41 (95.3) | 43 (95.6) | 115 (33.5) | 110 (32.4) | 189 (41.9) | 91 (39.9) | 65 (18.2) | 26 (14.5) |
| 1 | 2 (4.7) | 2 (4.4) | 227 (66.2) | 229 (67.4) | 261 (57.9) | 136 (59.6) | 293 (81.8) | 153 (85.5) |
| 2 | 0 | 0 | 1 (0.3) | 1 (0.3) | 0 | 1 (0.4) | 0 | 0 |
| Smoking History, n (%) |  |  |  |  |  |  |  |  |
| Never | 12 (27.9) | 8 (17.8) | 32 (9.3) | 23 (6.8) | 48 (10.6) | 17 (7.5) | 50 (14.0) | 20 (11.2) |
| Current/Former | 31 (72.1) | 37 (82.2) | 311 (90.7) | 316 (92.9) | 403 (89.4) | 211 (92.5) | 308 (86.0) | 159 (88.8) |
| Disease Stage, n (%) |  |  |  |  |  |  |  |  |
| IIIA/IIIB | 43 (100) | 45 (100) | - | - | - | - | 103 (28.8) | 49 (27.4) |
| IV | 0 | 0 | 343 (100) | 340 (100) | 451 (100) | 228 (100) | 255 (71.2) | 130 (72.6) |
| **Liver Metastases, n (%)** | **-** | **-** | 70 (20.4) | 69 (20.3) | 69 (15.3) | 31 (13.6) | 40 (11.2) | 17 (9.5) |
| **Brain Metastases, n (%)** | **-** | **-** | - | - | - | - | 20 (5.6) | 18 (10.1) |
| **Histology, n (%)** |  |  |  |  |  |  |  |  |
| Squamous | 27 (62.8) | 32 (71.1) | 0 | 0 | 0 | 0 | 353 (98.6) | 175 (97.8) |
| Non-Squamous | 16 (37.2) | 13 (28.9) | 343 (100) | 340 (100) | 451 (100) | 228 (100) | 5 (1.4) | 4 (2.2) |

**Abbreviations:** Atezo, Atezolizumab; Cam, Camrelizumab; Chemo, Chemotherapy; CnP, Carboplatin + nab-paclitaxel; ECOG PS, Eastern Cooperative Oncology Group Performance Status; Pla, Placebo; Serp, Serplulimab.

**Note:**"-" indicates that data was not reported or not applicable for the specific trial population; ECOG PS  "2" indicates that unknown ECOG PS.

*IMPower131 (Jotte et al.) and IMPower130 (West et al.) studied primarily non-squamous and exclusively non-squamous NSCLC, respectively. ASTRUM-004 (Zhou et al.) enrolled exclusively squamous NSCLC. The trial by Lei et al. included a mixed population.*
